# Clinical Comparison of the Subepithelial Connective Tissue versus Platelet-Rich Fibrin for the Multiple Gingival Recession Coverage on Anterior Teeth Using the Tunneling Technique

**DOI:** 10.1155/2017/4949710

**Published:** 2017-06-13

**Authors:** Victor Fabrizio Cabrera Pazmiño, Miguel Agusto Riquelme Rodas, Carlos David Barrios Cáceres, Guillermo Gustavo Renault Duarte, Melanie Vanesa Cano Azuaga, Bruna Luísa de Paula, Eliana Aparecida Caliente, Simone Soares, Elcia Maria Varize Silveira

**Affiliations:** ^1^Hospital for Rehabilitation of Craniofacial Anomalies (HRAC), University of São Paulo (USP), Bauru, SP, Brazil; ^2^Department of Oral Surgery, Universidade do Sagrado Coração (USC), Bauru, SP, Brazil; ^3^Department of Periodontics, Universidad del Pacífico (UP), Asunción, Paraguay; ^4^Department of Surgery, Universidad Católica Nuestra Señora de la Asunción-Guairá (UCG), Guairá, Paraguay; ^5^Universidad del Pacífico (UP), Asunción, Paraguay; ^6^Department of Surgery and Integrated Clinic, Araçatuba Dental School, Universidade Estadual Paulista (UNESP), Araçatuba, SP, Brazil; ^7^Department of Prosthodontics, Bauru School of Dentistry (FOB) and Hospital for Rehabilitation of Craniofacial Anomalies (HRAC), University of São Paulo (USP), Bauru, SP, Brazil; ^8^Department of Biological Sciences, Bauru School of Dentistry (FOB), University of São Paulo (USP), Bauru, SP, Brazil

## Abstract

The aim of this study was to evaluate and compare, clinically, the efficiency of the subepithelial connective tissue graft (SCTG) and platelet-rich fibrin (L-PRF) using the tunnel technique to cover the multiple gingival recessions on anterior teeth, in the same patient. Within the limits of this study, we conclude that both SCTG and L-PRF proved to be reliable options for the treatment of gingival recessions, efficiently supporting the biological and aesthetic demand, stimulating the periodontal tissues' health, and bringing reliable and highly predictable results.

## 1. Introduction

The plastic periodontal surgeries main goals are to improve the health, aesthetic, and regeneration of gingival and periodontal tissues [1]. Thus, the root coverage procedure to correct the keratinized mucosa variation level around the tooth surface which is caused by the gingival retraction has gained popularity over the past few years [2].

The gingival recession is defined by the pathological apical migration of the gingival margin in relation to the cementoenamel junction (CEJ), exposing the root surfaces [3] and bringing about functional and aesthetic problems, such as dentine hypersensitivity and root cavity, localized or widespread [4].

Among the surgical techniques which are consecrated in the literature to treat the gingival retraction [5, 6], the tunneling technique (TT), with coronary repositioned flap, advocates the integrity of the interdental papilla and the preservation of the tissue at issue, seeking to be less invasive [7]. This technique has been associated with subepithelial connective tissue graft (SCTG) in patients with multiple recession, with high success rates [8].

The use of the SCTG for the root coverage in periodontal surgery is considered the golden pattern to the gingival recession treatment [9, 10] due to its characteristics of quick keratinization and adherence. However, the application of the technique is limited by the thickness of the giver tissue, anatomical factors [11], technical difficulty, and the need of an additional giver site [1]. Due to these limitations, investigations on more regenerative nature techniques have been explored, such as the platelet-rich fibrin (L-PRF), to cover the gingival recessions [4, 11, 12].

The use of L-PRF membranes is easy to prepare and manipulate and they do not require the use of anticoagulant [13]; thus, they can be cut, adapted, and easily sutured [14]. The L-PRF, when used in deformities of recession, repairs the functional properties of the teeth's vestibular gingiva and restores the permanence and integrity of the keratinized gingival [15].

Considering the above, the report of the following case presents the clinical comparison of the subepithelial connective tissue graft (SCTG) versus platelet-rich fibrin (L-PRF) for multiple gingival recession coverage in anterior teeth, using the tunneling technique (TT).

## 2. Case Presentation

**Figure 1:**
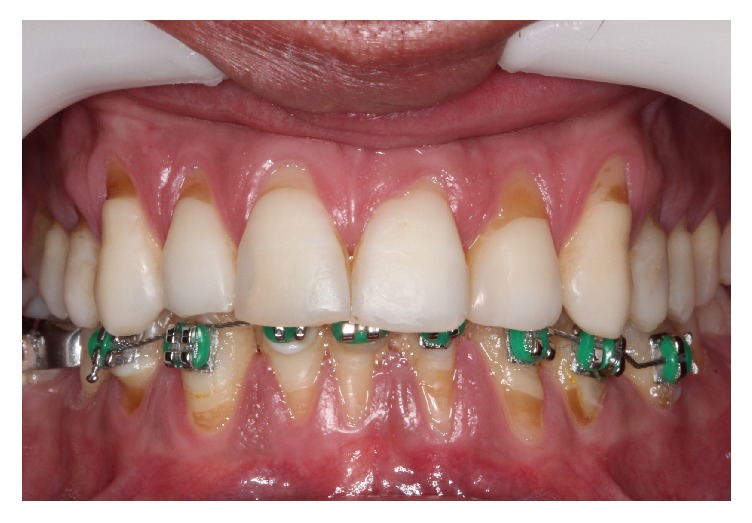
Patient with multiple Miller class II gingival recessions, on the vestibular face of the upper and lower teeth.

A male patient, 35 years old, smoker, presented himself at the periodontal clinic of Universidad del Pacífico (UP), Asunción, Paraguay, presenting high sensitivity in the anterior teeth and a longer aspect compared to the other ones. A clinical exam was carried out and multiple Miller's recessions class II was identified, in the vestibular phase in the upper and lower teeth (Figure 1). The suggested treatment was the TT execution with a coronally positioned flap, and the L-PRF membranes procedure was suggested for the exposed root coverage of the 11-12-13-14 teeth, while the SCTG was chosen for the root coverage of the 21-22-23-24 teeth with the purpose of finding out which coverage material would give the best aesthetic result and comfort in a short period of time.

### 2.1. L-PRF Obtaining

**Figure 2:**
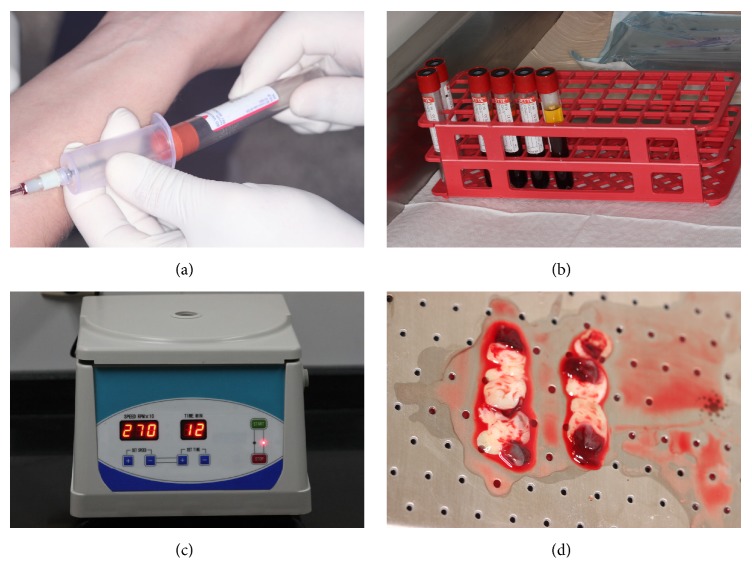
(a) Blood collection. (b) Blood stocking tubes. (c) Centrifugation. (d) Platelet-rich fibrin membranes.

Before the surgical procedure, a 40 ml venous blood collection was withdrawn from the patient (Figure 2(a)). The blood was stored in four tubes of 10 ml each (BD Vacutainer Serum, NJ, USA) (Figure 2(b)) and the samples were centrifuged at 2700 rpm for 12 minutes (Benchtop Low Speed Centrifuge DT5-6A, Hunan, China) (Figure 2(c)), to obtain the L-PRF clot, which was positioned and compressed on a sterile metallic surface in order to obtain the membrane shape (Figure 2(d)).

### 2.2. Preparation of the Root Surfaces

**Figure 3:**
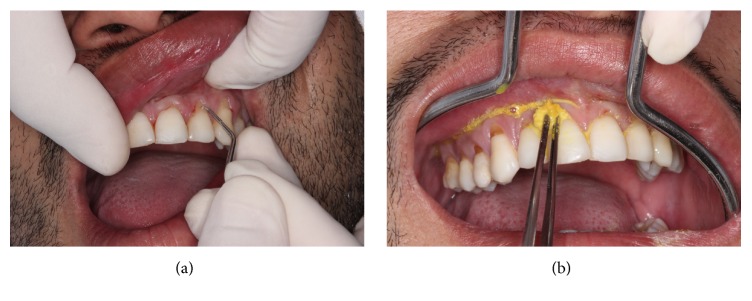
(a) Scraping and root smoothing. (b) Smear with tetracycline.

Soon after, the patient was guided to rinse the mouth for one minute using 15 ml of chlorhexidine solution on 0,12% and chlorhexidine solution on 2% for extraoral antisepsis. Afterwards, a local anesthesia was given using the infiltrated terminal technique making use of mepivacaine on 2% with epinephrine 1.100.00 (Mepiadre, Nova DFL Ind. e Com. SA Rio de Janeiro, RJ, Brazil), at the bottom of the vestibular-oral of the superior arch and on the hard palate, to the right side. Immediately, the exposed roots were cleaned up through scraping and root smoothing with periodontal curettes (Figure 3(a)); subsequently, they were treated and demineralized with tetracycline smear of 500 mg and dissolved in 10 ml of saline solution, using cotton during three minutes to remove the smear layer (Figure 3(b)).

### 2.3. Surgical Procedure

**Figure 4:**
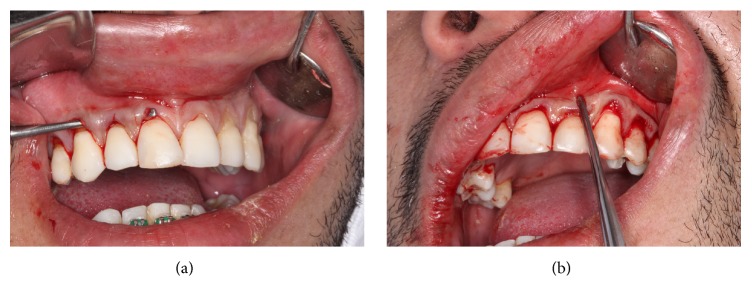
(a) Intrasulcular incisions and conservation of the interdental papillae. (b) Mucoperiosteal tunnel to release the partial thickness flap.

Using a scalpel blade 15C, intrasulcular incisions were taken in internal bezel in the vestibular face of the teeth 14-13-12-11-21-22-23-24, aiming to create an envelope of partial thickness, extending sideways until the distal of the second premolars and apically until overrunning the mucogingival line, including the interdental papilla without tearing them (Figure 4(a)). Afterwards, with the support of the periodontal tunnel makers (Hu-Friedy, Ipanema, RJ, Brazil), a mucoperiosteal tunnel was created to release the flap and the coverage of the recessions of each quadrant (Figure 4(b)).

### 2.4. Preparation of the Donor Site

**Figure 5:**
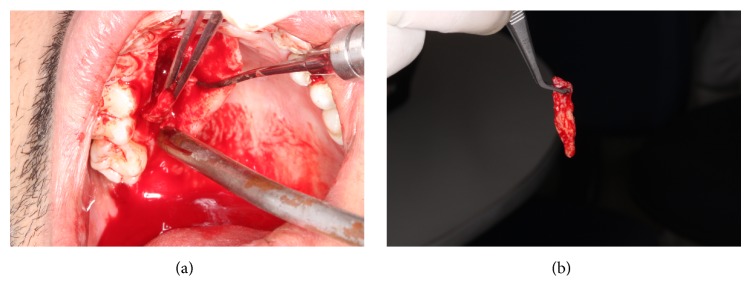
(a) Demarcation of the graft area on the donor site. (b) Removal of the subepithelial connective tissue.

Later on, a sterile portion of paper sheet was used to map the left side SCTG's recipient-site and then transferred to the region of the right side palate, delineating the graft in the donor site. Subsequently, the incisions were made: a perpendicular paracrestal incision along the teeth axis, at 3 mm from the palatine gingival contour, preserving the biological distance of the surrounding teeth (trap technique) (Figure 5(a)). A SCTG of around 24 mm × 7 mm and 2 mm of thickness was obtained and removed by using a fine extractor (Figure 5(b)).

### 2.5. Preparation of the SCTG's Recipient Site

**Figure 6:**
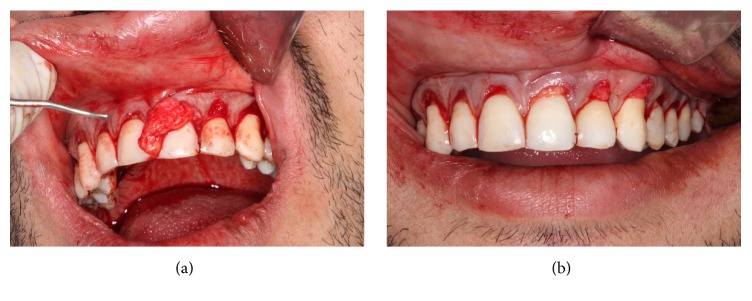
(a) Inserted graft on the recipient site. (b) Graft positioned on the recipient site.

**Figure 7:**
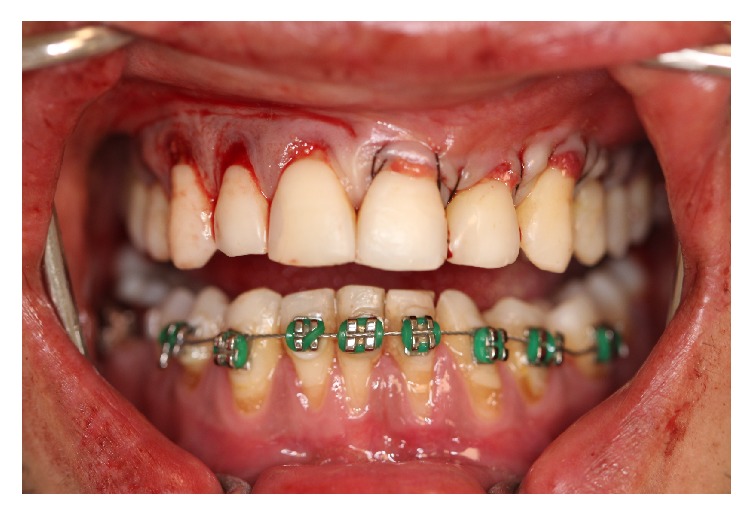
Stabilization and suture of the subepithelial connective tissue.

**Figure 8:**
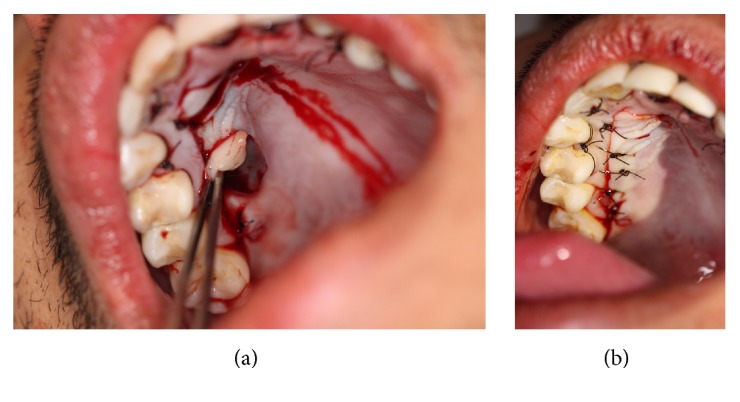
(a) Insertion of platelet-rich fibrin on the palate. (b) Simple suture on the donor site.

Thereafter, the graft was inserted and positioned (Figure 6(a)) covering the recessions in the recipient site in anteroposterior direction (Figure 6(b)); a light pressure was performed in the graft/flap complex with gauze for eight minutes in order to have a minimum clot thickness; besides, the stabilization of the SCTG was performed with suspensory and vertical mattress sutures (Figure 7), using nylon fiber 4-0 (Ethicon, Johnson & Johnson, São José dos Campos, SP, Brazil), in close contact with the periosteum. In the donor area of the palate, L-PRF membranes were inserted (Figure 8(a)), and, afterwards, simple sutures were performed by using nylon fiber 4-0 (Ethicon, Johnson & Johnson, São José dos Campos, SP, Brazil) to favor closure by primary intent (Figure 8(b)).

### 2.6. L-PRF Membrane Application

**Figure 9:**
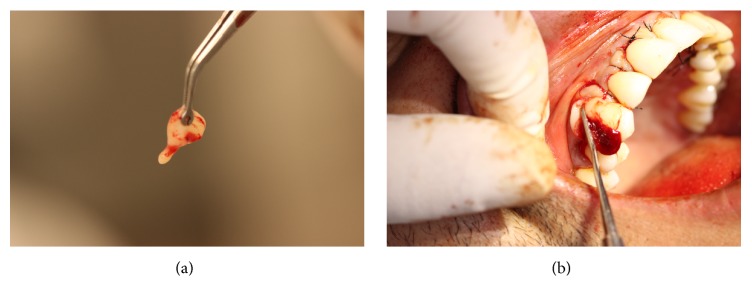
(a) Platelet-rich fibrin membranes. (b) Application of platelet-rich fibrin membranes on the recipient-site.

**Figure 10:**
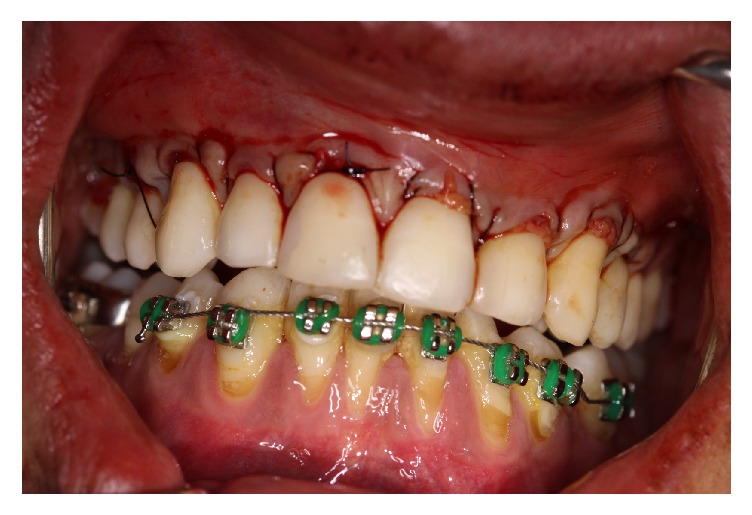
Stabilization and suture of the platelet-rich fibrin membranes.

To the right side, using a pair of modified tweezers, a plenty of L-PRF membranes were inserted and adapted, around 5 mm × 3 mm and 2 mm of thickness in each exposed root surface to recover the recessions (Figures 9(a) and 9(b)), which were obtained from clots resulting from centrifugation. The flap was positioned in coronal direction and maintained by suspensory and vertical mattress sutures in each tooth involved in the process (Figure 10).

### 2.7. Postsurgical Care

**Figure 11:**
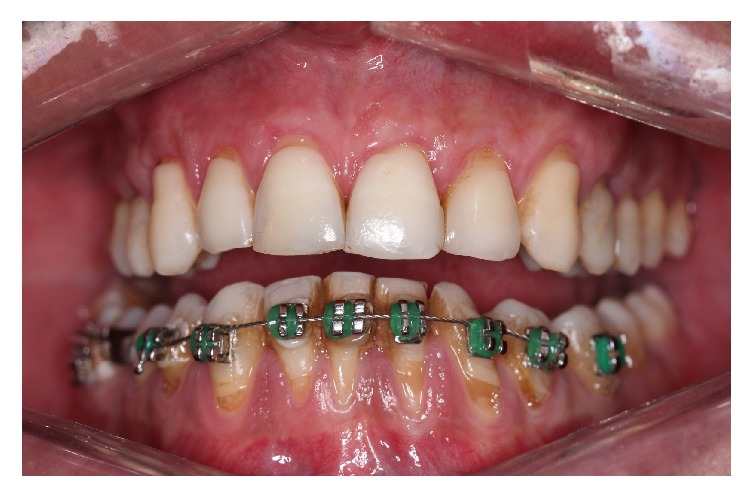
Stabilized mucogingival tissue with satisfactory appearance 45 days after the root coverage surgery.

After the surgery, 500 mg amoxicillin was prescribed, three times a day during seven days, 20 mg ketorolac three times a day during three days, 50 mg diclofenac three times a day during three days, besides rinsing with chlorhexidine gluconate 0,12% (Periogard, Colgate Palmolive) twice a day during two weeks. The sutures were removed after a week and the patient was instructed to have the oral hygiene using the proper tooth-brushing technique with soft-bristle toothbrush. The healing procedure came naturally and, after 45 days, a significant repairing and regeneration of the collagen fibers in the entire superior arch, great thickness and great quality of the mucogingival tissue, and satisfactory appearance were observed (Figure 11).

## 3. Discussion

The clinical case, which was presented in this article, describes the clinical comparison between the subepithelial connective tissue graft (SCTG) and platelet-rich fibrin (L-PRF) for the coverage of multiple gingival recessions on anterior teeth using the tunneling technique (TT). Due to Miller's class II recession, the patient had general hypersensitivity and out-of-proportion appearance of teeth, which compromised his physical appearance and nutrition.

In this case report, it is possible to notice the migration of the mucosa in relation to the mucogingival line in apical direction and a narrow keratinized gingiva strip. These modifications in the gingival mucosa level were related to a traumatic brushing and excessive orthodontic strength, which could have exacerbated the gingival recessions [16]. The TT was chosen because of the versatility and success predictability, since the sites with no or little keratinized gingiva and tenuous thickness do not represent absolute contraindication for its execution. This technique associated with the coronal positioning provided extreme security with minimum manipulation and graft tension, providing an excellent blood supply to the grafting materials. Ensuring less postoperative discomfort, this technique is indicated by literature for multiple gingival recession [7]. In addition to that, this technique favored the coverage of recessions in almost all of the exposed roots, increasing the thickness and extension of the keratinized tissue strip in the vestibular of the anterosuperior teeth.

An important factor for the success of root coverages is the treatment of the root surface in order to offer a proper condition while seeking the periodontal reconstruction [17]. The demineralization of the root surface with tetracycline for the conditioning of the exposed dental roots promoted the consolidation of the grafting materials proposed in this clinical case, and it helped the cellular proliferation and distinction [18]. This mechanical removal of the bacterial deposit from the root cement was important for the formation of the “new conjunctive attachment.”

The use of SCTG for the resolution of the recessions and the increasing of the keratinized gingiva strip, it is based on its excellent biomimetic capacity, highlighting the induction potential of two fundamental characteristics: the keratinization of the gingival mucosa and a new adhesion of periodontal connective tissue [8]. The SCTG associated with the TT provided a chromatic replication and original gingival texture, allowing us to reach satisfactory aesthetic results [19]. Besides, the blood supply to the grafted connective tissue was a key element in the TT for the creation of the graft tissue of partial thickness in the vestibular face, avoiding the exposure of the alveolar bone [8, 20].

In relation to the L-PRF, the coverage of the superior hemiarch is based on the property of growth factors found in the platelet of gel-rich fibrin and leukocyte [21], stimulating the angiogenesis, immunity, and epithelial proliferation through the formation of the initial fibrin clot [22]. The L-PRF membranes benefit the regeneration of the bone stimulating the stabilization of the initial fibrin clot [1] and accelerate the healing process. Furthermore, using the L-PRF, the need of a donor site was eliminated, making this technique less invasive, bringing about the reduction of the edema formation compared to the SCTG and less postsurgical discomfort [11].

Thus, a clinical evaluation was performed at the treated sites 45 days after the surgical procedures and the results showed that both SCTG and L-PRF membranes brought about satisfactory results of the Miller class II recession coverage, with 90% coverage of the exposed root, decreasing the dental hypersensitivity and reaching the patient expectations.

Within the limits of this study, it can be concluded that both SCTG and L-PRF brought about a significant quantity of root coverage and presented themselves as reliable options for the gingival recession treatment, effectively promoting the biological and aesthetic demands, stimulating the health of periodontal tissues, and bringing reliable and highly predictable results.

However, the tunneling technique resulted in less surgical time and was less invasive, resulting in less postoperative discomfort favoring better aesthetic results.
